# Arthroscopic rotator cuff repair with and without subacromial decompression is safe and effective: a clinical study

**DOI:** 10.1186/s12891-019-3032-z

**Published:** 2020-01-11

**Authors:** Umile Giuseppe Longo, Stefano Petrillo, Vincenzo Candela, Giacomo Rizzello, Mattia Loppini, Nicola Maffulli, Vincenzo Denaro

**Affiliations:** 1 Department of Orthopaedic and Trauma Surgery, Campus Bio-Medico University, Via Alvaro del Portillo, 200, 00128 Rome, Italy; 2 Centro Integrato di Ricerca (CIR) Campus Bio-Medico University, Via Alvaro del Portillo, 21, 00128 Rome, Italy; 3 Department of Biomedical Sciences, Humanitas University, Via Rita Levi Montalcini 4, 20090 Milan, Italy; 4 Department of Orthopaedic and Trauma Surgery, Humanitas Clinical and Research Center, Via Alessandro Manzoni 56, 20089 Milan, Italy; 5 Department of Musculoskeletal Disorders, Faculty of Medicine and Surgery, University of Salerno, Salerno, Italy; 6 Centre for Sports and Exercise Medicine, Barts and The London School of Medicine and Dentistry, Mile End Hospital, 275 Bancroft Road, London, E1 4DG England; 7 Keele University Faculty of Medicine, School of Pharmacy and Bioengineering, Guy Hilton Research Centre, Thornburrow Drive, Hartshill, Stoke-on-Trent, ST4 7QB England

**Keywords:** Rotator cuff, Subacromial decompression, Arthroscopy, Outcome

## Abstract

**Background:**

Subacromial decompression, that consists of the release of the coracoid-acromial ligament, subacromial bursectomy and anterior-inferior acromioplasty, has traditionally been performed in the management of this pathology. However, the purpose of subacromial decompression procedure is not clearly explained. Our reaserch aimed to analyse the differences among the outcomes of arthroscopic rotator cuff repair (RCR) made with suture anchors, with or without the subacromial decompression procedure.

**Methods:**

116 shoulders of 107 patients affected by rotator cuff (RC) tear were treated with Arthroscopic RCR. In 54 subjectes, the arthroscopic RCR and the subacromial decompression procedure (group A) were executed, whereas 53 took only arthroscopic RCR (group B). Clinical outcomes were evaluated through the use of the modified UCLA shoulder rating system, Wolfgang criteria shoulder score and Oxford shoulder score (OSS). Functional outcomes were assessed utilizing active and passive range of motion (ROM) of the shoulder, and muscle strength. The duration of the follow up and the configuration of the acromion were used to realize the comparison between the two groups.

**Results:**

In patients with 2 to 5 year follow up, UCLA score resulted greater in group A patients. In subjectes with longer than five years of follow up, group B patients showed considerably greater UCLA score and OSS if related with group A patients. In subjectes that had the type II acromion, group B patients presented a significant greater strength in external rotation.

**Conclusion:**

The long term clinical outcomes resulted significantly higher in patients treated only with RCR respect the ones in patients underwent to RCR with subacromial decompression.

## Background

The prevalence of rotator cuff (RC) lesions chenges between 5 to 39% among people, often occurring in patients younger than 40 years old, and may be bilateral in 16% of patients [1–5]. Outcomes of surgical repair are unpredictable, in part because surgical repair does not reproduce the natural enthesis, and in part because the physiological processes responsible for the ability of the tendon to reconnected to the bone have not been completely known [6–13].

Subacromial impingement syndrome was considered the most frequent cause of RC pathology, and Neer [1, 14, 15] was the first that described this condition as an irritation of the subacromial tissue under the coraco-acromial arch with consequent degeneration and rupture. The role, and even the presence, of subacromial impingement has recently been questioned [16]. Spurs on the acromion and the shape of the acromion are often associated with RC tears, even if their causality has not been proved [16, 17].

Subacromial decompression that comprehend the release of the coracoid-acromial ligament, subacromial bursectomy and anterior-inferior acromioplasty has traditionally been performed in the management of this pathology. Various researches have shown excellent results of arthroscopic RC repair (RCR) conducted together with subacromial decompression [18, 19]. However, only one randomized controlled trial has compared the arthroscopic RCR outcome realized with or without the subacromial decompression [20].

Our research aims to analyse the differences between clinical and functional outcomes of arthroscopic RCR with or without subacromial decompression in patients with full-thickness RC tears.

## Methods

### Ethics

The ethics committee of the university “Campus Bio Medico” of Rome authorized the research and all subjects signed consent to be enrolled.

### Patient enrolment

Inclusion criteria were: RC tear, absence of shoulder instability, absence of fracture of the higher/lower tuberosity of the humerus or the glenoid, full-thickness RC tear confirmed by shoulder MRI, pain and/or functional impairment of the shoulder for at least six months, symptoms refractory to conservative protocol of treatment (anti-inflammatory, rehabilitation, repose, and local corticosteroid infiltration), treatable RC tears.

Exclusion criteria included: inflammatory joint disease, preceding operative treatment on the damaged shoulder, labral pathology agreeable to operative treatment, degenerative osteoarthritis of the glenohumeral joint, symptomatic osteoarthritis of the acromioclavicular joint, RC arthropathy, impossibility to write up questionnaires consequent to language disease or cognitive disturbs.

117 subjects (126 shoulders) presented the inclusion\exclusion criteria and were contacted telephonically. Of these, 107 (116 shoulders) accepted our invitation to participate to the study and were retrospectively examined and assigned into Group 1 (54 patients (59 shoulders) RCR associated with simultaneous subacromial decompression) or Group 2 (53 patients (57 shoulders) RCR without subacromial decompression). The two groups were evaluated in relation to the of clinical and functional outcomes according to the shape of acromion and length of the follow-up.

### Clinical assessment

Clinical outcomes were assessed employing the modified UCLA score, Wolfgang criteria, and Oxford Shoulder Score (OSS) score, all administered by two blinded examiners at the last follow-up.

The modified UCLA [21] evaluates post-surgical shoulder discomfort (10 points), function (10 points), active forward flexion (5 points), strength (5 points) and patient well-being (5 points). The final result extends from 0 to 35, and it can be subdivided in excellent (34–35 points), good (28–33), fair (21–27), or poor (0–20).

The Wolfgang criteria estimate post-operative shoulder discomfort (4 points), active abduction (4 points), strength (4 points) and patient well-being (1 point or minus 1 point). The final score extends from 0 to 17, and it can be subdivided in excellent (14–17 points), good (11–13 points), fair (8–10 points) or poor (0–7 points).

The OSS [22, 23] is a simple questionnaire made of 12 self-administered items assessing shoulder function, pain and strength related to quotidian actions. The final result ranges from 12 to 60 points, where 60 indicates the worst function of the shoulder.

### Functional assessment

Two blinded testers evaluated passive and active range of motion (ROM), and muscle strength at the last follow-up.

Standard measurement guidelines were used to assess supine passive and active forward elevation, intra and extra rotation ROM (90° abduction). A conventional goniometer was employed in order to calculate with scales scored in one-degree increments. A dynamometer (model 01163, Lafayette Instrument Company, Lafayette, Indiana) was utilized to estimate the strength of anterior elevation, intra and extra rotation of the shoulder.

Both testers made three estimations for each ROM and strength measurement examined. The average value for each variable was utilized for statistical determinations.

### Imaging

Preoperative MRI scans and oblique coronal, oblique sagittal and axial T2-weighted spin-echo MRIs (repetition time (RT) were executed for each subject: 3200 milliseconds; echo time (ET): 85 milliseconds) and standard radiographs (antero-posterior view, lateral view of the scapula, and axillary one).

### Arthroscopic technique

Patient were positioned in lateral decubitus: the brachial plexus block, together with the general anaesthesia, were performed. The arm was suspended at 45° of abduction and 20° of forward flexion with 4.5 to 6.5 kg of traction.

The RC back was mobilized to its bone site of attachment employing a lateral portal and the footprint of the greater tuberosity was weared. One row of suture anchors double loaded with N° 2 Fiberwire (Corkscrew or Biocorkscrew, Arthrex, Naples, FL) were positioned in the lateral aspect of the footprint to perform the RC repair. The subacromial decompression, that consist of the subacromial bursectomy, the release of coraco-acromial ligament and of the antero-inferior acromioplasty was performed in all patients of Group 1, often because of acromion type III or acromial spurs or reduced subacromial space or acromioclavicular joint arthritis. In acromion type III, the most anterior portion of the acromion has a hooked shape with a subacromial spur. However, subacromial decompression was also performed in patients with subacromial spurs and without acromion type III.

### Postoperative management

Post-surgical protocol was identical for the two categories of patients. An abduction bolster was used for 6 weeks. Active elbow flexion and extension were permitted, but the last part of the extension and overhead stretching was limited. The day immediately after the surgery patients began passive external rotation of the arm. The sling was removed after 6 weeks. Isoinertial strengthening and physiotherapy of the rotator cuff, deltoid and scapular were started at 10–12 weeks then surgery. Rehabilitative Physiotherapy was prolonged for 6 months. Substantial manual task and overhead activities were permitted 6 to 10 months post surgical treatment.

### Sample size and power analysis

It was made a post hoc power analysis on our results with a specific statistical software (G*Power, version 3.1.2, Heinrich Heine University, Dusseldoerf, Germany). According to the post hoc power analysis, our study power to detect a significant difference is of 0.80 with and an *alfa* error of 0.05.

### Statistics

The Statistics was elaborated utilizing the clinical and functional outcome scores. Then we studied active and passive ROM and strength of shoulder range of movement. The independent variables analyzed were: age; gender; arm dominance; history of trauma; size of the rotator cuff tear; biceps tendon break or tendinopathy; type of treatment of biceps tendon; configuration of the acromion. A two-tailed Mann-Whitney U test for continuous variables and the X2 test for categorical variables were used to evaluate each independent variable for the two patients groups. The outcome variables examined were analyzed using a two-tailed Mann-Whitney U test. The level of significativity was fixed at *P* < 0.05.

## Results

### Demographics

Demographic data and surgical details of the enrolled subjects are reported in Table 1. we noticed no significative differences among the groups in terms of age, gender, arm dominance, history of trauma, size of lesion, and shape of the acromion.

**Table 1:** Demographic and surgical details of the enrolled patients

VARIABLES	GROUP A (pts = 54, s = 59)	GROUP B (pts = 53, s = 57)	*P* value
Age mean + SD (range)	59 + 10 (range 29 to 76)	56.2 + 8.5 (range 29 to 70)	0.05
Gender male:female	24:30	25:28	0.46
Arm dominance dominant:nondominant	44:15	45:12	0.5
Trauma traumatic:degenerative	13:46	12:45	0.86
Size of lesion non massive:massive	39:20	44:13	0.08
LHB pathology			
none	34	19	0.02*
tear	17	24	0.03*
tendinophaty	8	14	0.05
LHB management			
none	29	24	0.32
tenotomy	23	21	0.77
tenodesis	7	12	0.07
Acromion shape			
I	25	28	0.32
II	26	22	0.38
III	8	7	0.83

*LHB* long head of the biceps; *SD* standard deviation; *pts* patients; *s* shoulders

* statistically significant

### Clinical assessment

All patients had a follow up of at least 5 years. The comparison among the two patient categories respect to the duration of follow up did not show any significative difference for each questionnaire administered at 2 years. Between 2 to 5 year of follow up, the UCLA score was greater in the patients who underwent subacromial decompression. On the other hand, at five years of follow up, the group without subacromial decompression shown UCLA score significantly greater and OSS significantly lesser when equated with the group which underwent subacromial decompression **(**Table 2**)**.

**Table 2:** Clinical outcomes of the enrolled patients according to the follow up

FOLLOW UP	< 2 yrs			2–5 yrs			> 5 yrs		
OUTCOMES SCORES	G A mean + SD (range)	G Bmean + SD (range)	*P* value	G A mean + SD (range)	G B mean + SD (range)	*P* value	G A mean + SD (range)	G B mean + SD (range)	*P* value
UCLA									
Pain	5.8 + 2.8 (1–10)	6.8 + 2.7 (1–10)	0.39	6.9 + 2.4 (1–10)	6.4 + 2.8 (1–10)	< 0.0001*	6.8 + 2.5 (2–10)	7.8 + 2.7 (2–10)	0.18
Function	6.6 + 3 (2–10)	7.3 + 3 (1–10)	0.5	7.1 + 2.4 (2–10)	6.3 + 3 (1–10)	< 0.0001*	6.6 + 2.2 (2–10)	8.4 + 2.7 (2–10)	0.02*
Forward Flexion	4.2 + 1 (2–5)	4.5 + 0.9 (2–5)	0.37	4.4 + 0.9 (2–5)	4.4 + 0.8 (3–5)	< 0.0001*	4.5 + 0.9 (2–5)	4.5 + 1 (1–5)	0.69
Strength	3.8 + 1.2 (1–5)	3.8 + 1.1 (2–5)	0.98	3.4 + 1 (1–5)	3.7 + 1 (2–5)	< 0.0001*	3.7 + 0.7 (3–5)	4.2 + 0.7 (3–5)	0.09
Satisfaction	5 + 0	4.2 + 1.8 (0–5)	0.5	4.8 + 0.9 (0–5)	4.8 + 1 (0–5)	0.002*	4.6 + 1.3 (0–5)	5 + 0	0.74
*Final score*	25.4 + 7.5 (11–35)	26.8 + 8.4 (6–35)	0.53	26.6 + 6 (6–35)	25.6 + 7.45 (10–35)	< 0.0001*	26.3 + 5.8 (15–34)	29.9 + 6.2 (15–35)	0.04*
WOLFGANG									
Pain	2.8 + 0.8 (1–4)	2.8 + 1.2 (0–4)	0.81	2.9 + 0.8 (1–4)	2.6 + 1.1 (1–4)	0.42	2.9 + 0.8 (1–4)	3.2 + 0.7 (2–4)	0.45
Active abduction	3.4 + 0.7 (2–4)	3.5 + 1.1 (1–4)	0.33	3.3 + 1 (1–4)	3.3 + 0.9 (2–4)	0.96	3.5 + 0.9 (1–4)	3.7 + 0.7 (2–4)	0.49
Strength	3 + 0.9 (1–4)	3.1 + 0.9 (1–4)	0.78	3 + 0.7 (2–4)	3.1 + 0.9 (1–4)	0.24	3 + 0.7 (2–4)	3.5 + 0.6 (2–4)	0.11
Function	3.2 + 0.8 (2–4)	3.2 + 0.9 (1–4)	0.81	3 + 0.6 (1–4)	3.3 + 0.8 (1–4)	0.72	3 + 0.5 (2–4)	3.4 + 0.5 (3–4)	0.12
Satisfaction	0.8 + 0.7 (− 1–1)	0.7 + 0.7 (− 1–1)	0.91	0.9 + 0.4 (− 1–1)	0.9 + 0.4 (− 1–1)	0.84	0.9 + 0.5 (− 1–1)	1 + 0	0.73
*Final score*	13.2 + 2.9 (7–17)	13.3 + 4.3 (2–17)	0.59	13 + 2.7 (4–17)	13.2 + 3.6 (6–17)	0.66	13.4 + 2.4 (8–17)	14.8 + 2 (10–17)	0.07
OSS	26.1 + 12.7 (12–51)	22.8 + 10.7 (12–47)	0.47	21.9 + 9.8 (12–53)	25.7 + 12.3 (12–51)	0.34	22.5 + 10.6 (12–43)	18.6 + 7.2 (12–32)	0.03*

*G A* group with subacromial decompression; *G B* group without subacromial decompression; *SD* standard deviation; *UCLA* University of California Los Angeles; *OSS* Oxford Shoulder Score

* statistically significant

Regarding the shape of the acromion, no statistically significative differences were found for each questionnaire administered. **(**Table 3**)**.

**Table 3:** Clinical outcomes of the enrolled patients according to the shape of acromion

TYPE OF ACROMION	I			II			III		
OUTCOMES SCORES	G A mean + SD (range)	G B mean + SD (range)	*P* value	G A mean + SD (range)	G B mean + SD (range)	*P* value	G A mean + SD (range)	G B mean + SD (range)	*P* value
UCLA									
Pain	6.8 + 2.5 (1–10)	6.9 + 3.2 (1–10)	0.62	6.5 + 2.6 (2–10)	7.3 + 2.2 (2–10)	0.3	6.1 + 3.1 (1–10)	6.7 + 3.5 (1–10)	0.78
Function	7.3 + 2.4 (2–10)	7.1 + 3.3 (1–10)	0.74	6.4 + 2.8 (2–10)	7.9 + 2.7 (2–10)	0.03*	6.7 + 2.4 (2–10)	6.1 + 3.8 (1–10)	0.95
Forward Flexion	4.5 + 0.9 (2–5)	4.5 + 0.9 (3–5)	0.6	4.3 + 0.9 (2–5)	4.5 + 1 (1–5)	0.1	4.1 + 1.4 (2–5)	4.3 + 1.1 (2–5)	1.00
Strength	3.5 + 1.1 (1–5)	3.9 + 1 (2–5)	0.3	3.7 + 1 (2–5)	4.1 + 0.9 (2–5)	0.15	3.2 + 1.3 (1–5)	3.4 + 1 (2–5)	1.00
Satisfaction	4.8 + 1 (0–5)	4.8 + 1 (0–5)	1.00	5 + 0	4.8 + 1 (0–5)	0.29	4.4 + 1.8 (0–5)	4.3 + 1.9 (0–5)	0.95
*Final score*	27.1 + 6.6 (6–35)	27.3 + 8.2 (10–35)	0.41	25.8 + 6.1 (15–35)	28.6 + 5.9 (13–35)	0.09	24.6 + 7.7 (11–33)	24.9 + 10 (6–35)	0.87
WOLFGANG									
Pain	3.1 + 0.8 (1–4)	3 + 1 (1–4)	0.84	2.7 + 0.9 (1–4)	2.8 + 0.9 (1–4)	0.81	2.6 + 0.7 (1–3)	2.7 + 1.6 (0–4)	0.46
Active abduction	3.5 + 0.9 (1–4)	3.4 + 0.9 (2–4)	0.81	3.3 + 0.8 (1–4)	3.7 + 0.8 (1–4)	0.02*	3.2 + 1.2 (1–4)	3.1 + 1.2 (1–4)	0.87
Strength	3.2 + 0.6 (2–4)	3.3 + 0.9 (1–4)	0.28	3 + 0.7 (2–4)	3.4 + 0.6 (2–4)	0.04*	2.5 + 1.1 (1–4)	2.9 + 1.1 (1–4)	0.54
Function	3 + 0.8 (1–4)	3.3 + 0.8 (1–4)	0.11	3.2 + 0.5 (2–4)	3.4 + 0.6 (2–4)	0.17	3 + 0.5 (2–4)	3.1 + 1.1 (1–4)	0.46
Satisfaction	0.8 + 0.7 (− 1–1)	0.9 + 0.4 (− 1–1)	0.28	1 + 0	0.9 + 0.4 (− 1–1)	0.29	0.7 + 0.7 (− 1–1)	0.7 + 0.7 (− 1–1)	0.95
*Final score*	13.5 + 3 (4–17)	13.9 + 3.4 (6–17)	0.33	13.2 + 2.2 (9–17)	14.2 + 2.6 (8–17)	0.09	12.1 + 3.2 (7–16)	12.6 + 5.4 (2–17)	0.4
OSS	23.1 + 11.7 (12–53)	21.9 + 12.4 (12–51)	0.32	21.6 + 9.2 (12–46)	21 + 7.4 (12–35)	0.94	28.5 + 14.3 (13–51)	28 + 13.6 (12–47)	0.78

*G A* group with subacromial decompression; *G B* group without subacromial decompression; *SD* standard deviation; *UCLA* University of California Los Angeles; *OSS* Oxford Shoulder Score

* statistically significant

### Functional assessment

Inter-group comparison according to the length of follow up did not display any significative difference in both passive and active ROM, and strength at each follow up time **(**Table 4**)**.

**Table 4:** Functional outcomes of the enrolled patients according to the follow up

FOLLOW UP	< 2 yrs			2–5 yrs			> 5 yrs		
OUTCOMES SCORES	G A mean + SD (range)	G B mean + SD (range)	*P* value	G A mean + SD (range)	G B mean + SD (range)	*P* value	G A mean + SD (range)	G B mean + SD (range)	*P* value
PASSIVE ROM(°)									
Anterior elevation	168.4 + 25.6 (90–180)	168.3 + 24.1 (110–180)	0.8	172.1 + 17.6 (100–180)	167 + 28.3 (90–180)	0.98	171.3 + 20.3 (120–180)	176.8 + 8.7 (140–180)	0.84
Extrarotation	69.4 + 22.6 (30–90)	72.1 + 20.1 (30–90)	0.73	76.8 + 17.7 (30–90)	70.4 + 23.5 (20–90)	0.45	65.7 + 33 (0–90)	83.3 + 20.1 (6–90)	0.42
Intrarotation	86.3 + 10.5 (50–90)	79.2 + 20.6 (30–90)	0.54	80.5 + 17.5 (30–90)	82.6 + 16.7 (30–90)	0.43	84 + 19.9 (10–90)	83.9 + 16.1 (30–90)	0.98
ACTIVE ROM (°)									
Anterior elevation	165.3 + 26.3 (90–180)	163.3 + 29.2 (100–180)	0.94	156.4 + 29.4 (90–180)	165.3 + 26.7 (90–180)	0.37	159.3 + 29 (90–180)	170.1 + 21.9 (100–180)	0.12
Extrarotation	43.4 + 13.2 (30–70)	40.4 + 15.3 (15–70)	0.73	47.5 + 19 (20–90)	43.3 + 20.1 (0–90)	0.59	41.6 + 16.3 (10–80)	48.6 + 15.7 (15–90)	0.26
STRENGHT (N)									
Anterior elevation	49.98 + 25.48 (14.7–107.8)	49 + 21.56 (19.6–83.3)	0.8	38.22 + 20.58 (9.8–107.8)	49 + 26.46 (19.6–107.8)	0.27	39.2 + 13.72 (19.6–68.6)	46.06 + 20.58 (19.6–107.8)	0.4
Extrarotation	48.02 + 24.5 (14.7–102.9)	49.98 + 21.56 (19.6–73.5)	0.91	43.12 + 18.62 (16.66–102.9)	50.96 + 21.56 (19.6–7.35)	0.32	36.26 + 11.76 (19.6–58.8)	47.04 + 19.6 (24.5–107.8)	0.12
Intrarotation	69.58 + 29.4 (24.5–107.8)	68.6 + 33.32 (9.8–107.8)	0.98	60.76 + 23.52 (29.4–107.8)	70.56 + 31.36 (29.4–107.8)	0.48	62.72 + 19.6 (29.4–107.8)	77.42 + 23.52 (39.2–11)	0.35

*G A* group with subacromial decompression; *G B* group without subacromial decompression; *SD* standard deviation

Inter-group comparison according to the shape of the acromion did exhibit any significative difference for both passive and active ROM, and strength in subjects with acromion type I and III according to Bigliani’s classification. In subjects with acromion type II, the group without subacromial decompression displayed a significantly greater strength in extrarotation **(**Table 5**)**.

**Table 5:** Functional outcomes of the enrolled patients according to the shape of acromion

TYPE OF ACROMION	I			II			III		
OUTCOMES SCORES	G A mean + SD (range)	G B mean + SD (range)	*P* value	G A mean + SD (range)	G B mean + SD (range)	*P* value	G A mean + SD (range)	G B mean + SD (range)	*P* value
PASSIVE ROM (°)									
Anterior elevation	169.6 + 24.2 (90–180)	168.9 + 26.5 (90–180)	0.92	174.4 + 14.7 (130–180)	173.5 + 17.5 (110–180)	0.59	163.7 + 27.2 (120–180)	171.4 + 22.7 (120–180)	0.54
Extrarotation	70.2 + 24.7 (20–90)	76.5 + 20 (30–90)	0.48	75.2 + 18.9 (30–90)	78.4 + 23.4 (30–90)	0.65	66.9 + 38.6 (0–90)	63.6 + 27.5 (20–90)	0.69
Intrarotation	84.6 + 12.5 (45–90)	79 + 20.3 (30–90)	0.45	85.2 + 14.4 (30–90)	86.1 + 13.7 (30–90)	0.51	70.6 + 30 (10–90)	82.3 + 18.9 (40–90)	0.54
ACTIVE ROM (°)									
Anterior elevation	159.2 + 29.1 (90–180)	163.7 + 29.9 (90–180)	0.33	165.2 + 24.9 (90–180)	170.9 + 19.7 (100–180)	0.56	142.5 + 37.3 (90–180)	167.1 + 29.9 (100–180)	0.23
Extrarotation	46 + 16.8 (20–80)	42.4 + 20 (0–90)	0.52	44 + 16.7 (20–90)	50.9 + 15.3 (20–90)	0.51	44.4 + 22.3 (10–90)	33.6 + 10.7 (15–45)	0.19
STRENGHT (N)									
Anterior elevation	39.2 + 16.66 (14.7–78.4)	43.12 + 20.58 (19.6–83.3)	0.75	44.1 + 23.52 (9.8–107.8)	51.94 + 24.5 (19.6–107.8)	0.23	40.18 + 28.42 (19.6–107.8)	48.02 + 30.38 (19.6–107.8)	0.46
Extrarotation	41.16 + 15.68 (16.66–68.6)	43.12 + 19.6 (19.6–7.35)	0.87	44.1 + 21.56 (14.7–102.9)	56.84 + 21.56 (19.6–107.8)	0.04*	42.14 + 26.64 (24.5–102.9)	44.1 + 17.64 (19.6–63.7)	0.54
Intrarotation	59.78 + 23.52 (29.4–107.8)	68.6 + 31.36 (29.4–107.8)	0.53	69.58 + 25.48 (24.5–107.8)	74.48 + 29.4 (9.8–107.8)	0.46	52.92 + 25.48 (29.4–107.8)	60.76 + 24.5 (29.4–98)	0.54

*G A* group with subacromial decompression; *G B* group without subacromial decompression; *SD* standard deviation

* statistically significant

### Complications

No patient developed complications or clinical symptoms of RC re-tears.

## Discussion

The principal finding of our job is that no significative difference respect to clinical outcome scores and functional outcomes revealed among subjects treated by RCR with or without subacromial decompression in every types of acromion by Bigliani’s classification. Moreover, according to the length of follow up, subjects who took RCR without subacromial decompression exhibited significantly superior long term clinical outcomes when compared with patients took by RCR with subacromial decompression.

Subacromial conflict syndrome is characterized by reduction of the subacromial space and alteration of the coraco-acromial arch [24–26]. It determines an irritation of the subacromial tissue with a major risk to develop a RC tear.

Morrison and Bigliani described three acromial shapes on outlet view radiographs (type I: flat undersurface; type II: curved; and type III: hooked) that raised with age and were related with RC tears. Several authors subsequently analyzed the possible relation between acromion morphologies and outcome after subacromial decompression. Gartsman and O’Connor showed that, in subjects with complete rotator cuff tear and acromion type II, acromioplasty did not bring to superior functional outcome [19]. Milano et al. [27], MacDonald et al. [28] and Abrams et al. [29] showed that acromion type exerted no significant influence on post-surgical functional scores. However, Abrams et al. [29] stated that a type III acromion harmed the Constant score, SST score, and VAS confronted with a type I acromion. Some authors suggested different causes related to the theory of impingement responsible of RC disease [16, 30]. Subacromial decompression is frequently performed in association with RCR. This procedure was effective in improving subacromial space, reducing subacromial tissue irritation, relieving pain and improving shoulder function [18, 31, 32]. However, no clinical studies have compared surgical issues with turning the acromion shape to a type 1 [16]. Moreover, several aspects of subacromial decompression as well as its role in association with RCR are still confusing or not well established.

The role of coraco-acromial ligament release is still debated, because it determines the destruction of coraco-acromial arch and may lead to the loss of active gleno-humeral elevation [33]. Furthermore, the release of the coraco-acromial ligament made together with acromioplasty determines the loss of secondary stabilizer of the humeral head against antero-superior migration and the consequent risk to develop rotator cuff pathology.

Indeed, the coracoacromial ligament was demonstrated to be the primary obstacle to superior dislocation of the humeral head, mainly if a large to massive RC lesion is present [34]. Finally, the current arthroscopic subacromial decompression technique generally recommends, if possible, the preservation of the coraco-acromial ligament [18, 31].

Acromioplasty is associated with a relevant percentage of satisfactory results [35, 36], and also massive and irreparable tears. However, its role is not clearly understood. In a nine-year follow up study of 96 subjects who took anterolateral acromioplasty, the RC disease progressed in 20% of them. The authors suggested other etiologic factors as causative of the condition. Another 9 year follow up cohort study found that more than one third of the subjects had RC disease advancement after anterolateral acromioplasty [37]. Anterolateral acromioplasty did not inhibit the advancement of the RC disease. In a systematic review of level I and level II studies, the authors made a comparison between the results of subacromial bursectomy with or without acromioplasty, concluding that, even though few high level randomized trials are available in the literature, similar results are obtained with subacromial bursectomy with or without acromioplasty [31]. In a systematic review and meta-analysis performed on four level I randomized studies including 373 patients, no significative differences in personal outcome successively to the arthroscopic RCR with or without acromioplasty at intermediate follow up were found [18]. Another level I randomized clinical trial, conducted on 120 patient with small or medium size rotator cuff tears, demonstrated that there were no significant variations concerning pain, ability of action, function and strength when comparing two groups of subjects underwent to RCR with or without acromioplasty [38]. These findings, therefore, suggest that acromioplasty could be no needful in the operative management of subjects with small- to medium-sized rotator cuff tears in lack of acromial spurs. Nevertheless, RCR failures are more frequently associated with other circumstances such as age, size of the RC tear, fatty degeneration of the tendons and intrinsic overload.

According to our results, significative differences about clinical and functional outcomes comparing arthroscopic RCR with or without subacromial decompression regardless of the shape of the acromion weren’t found. Moreover, patients without subacromial decompression exhibited superior clinical long term outcomes. For this reason, we believe that subacromial decompression procedure should be performed only in selected patients. It is possible that the worse outcome after spur resection was caused by degenerative changes of the tendon from the spur.

The primary point of strength of our study is that the two groups were homogeneous for all variables considered. Other forces are that two adequately prepared shoulder specialists made all the operations; two blinded examiners performed the follow-up evaluations and estimation of ROM and strength according to conventional guidelines.

We are conscious of our study limitations. First of all, the allocation of patients was not randomized, and the data were collected in a retrospective fashion; moreover, most of the patients allocated in the group 1 had a reduction of subacromial space because of an acromion type III or acromial spurs or acromioclavicular joint arthritis, and for this reason they underwent a subacromial decompression; we did not conduct an a precedent power analysis and sample size estimation, and we enrolled all the suitable subjectes trained in our hospital throughout the index period. However, the post hoc power analysis showed that the enrolled sample provided adequate power to justify the clinical relevance of our results.

## Conclusion

The long term clinical outcomes resulted significantly higher in patients treated only with RCR respect the ones in patients underwent to RCR with subacromial decompression.

## Data Availability

The datasets used and/or analysed during the current study available from the corresponding author on reasonable request.

## Abbreviations

MRI: Magnetic Resonance Imaging
N: Newton
OSS: Oxford shoulder score
RC: Rotator cuff
RCR: Rotator cuff repair
ROM: Range of motion
UCLA: University of California at Los Angeles
